# Clinical characteristics and surgical outcomes of full-thickness abdominal wall injuries complicated by evisceration and fistulation in dromedary camels (*Camelus dromedarius)*

**DOI:** 10.3389/fvets.2026.1926549

**Published:** 2026-09-29

**Authors:** El-Sayed El-Shafaey, Mahmoud Najeb, Mohamed Abdo Rizk, Mahmoud G. El Sebaei

**Affiliations:** 1 Department of Surgery, Anaesthesiology and Radiology, Faculty of Veterinary Medicine, Mansoura University, Mansoura, Dakahlia, Egypt; 2 Department of Veterinary Surgery, Salam Veterinary Group, Buraydah, Qassim, Saudi Arabia; 3 Department of Clinical Sciences, College of Veterinary Medicine, King Faisal University, Al-Ahsa, Saudi Arabia; 4 Department of Biomedical Sciences, College of Clinical Pharmacy, King Faisal University, Al-Ahsa, Saudi Arabia

**Keywords:** abdominal wall injuries, camels, evisceration, fistula, surgical management

## Abstract

**Background:**

Abdominal wall injuries in dromedary camels often result in life-threatening evisceration or persistent fistulation. While well documented in equines and bovines, standardized surgical management protocols and survival outcomes for camelids remain scarce in veterinary literature. This retrospective study evaluated the clinical patterns, visceral involvement, surgical treatments, and survival outcomes of full-thickness abdominal wall injuries complicated by evisceration or fistulation in dromedary camels.

**Methods:**

A retrospective study was conducted on 14 dromedary camels presenting with full-thickness abdominal wall trauma complicated by acute evisceration (*n* = 11) or chronic fistulation (*n* = 3) at a specialized camel hospital between September 2023 and December 2025. All animals underwent detailed clinical and ultrasonographic examinations. Surgical management was tailored according to lesion type, tissue viability, and the degree of contamination.

**Results:**

Females (85.7%) and the Wadeh breed (78.6%) were predominantly affected. Injury distribution was equally split between ventral/umbilical (50.0%) and ventrolateral (50.0%) zones, driven by external trauma (57.1%), hernia rupture (28.6%), or gastroschisis (14.3%). The intestines were the most frequently involved viscera (50.0%), and gross environmental contamination was present in 78.6% of cases. One camel (7.1%) died during initial stabilization. Following surgery of acute evisceration cohort, (63.6%) (7/11) survived and were successfully discharged from the hospital. Conversely, 27.3% (3/11) of the camels developed rapid hemodynamic collapse and hyperacute systemic sepsis within 24–48 h post-injury, which resulted in death. Postoperative recovery and successful hospital discharge of chronic fistulation were achieved in 66.7% (2/3) of the cases. One camel (33.3%, 1/3) developed uncontrollable severe sepsis secondary to chronic focal necrosis on postoperative day 4 and died.

**Conclusion:**

Accurate rapid diagnosis and prompt surgical interventions of full-thickness abdominal wall injuries complicated by evisceration and fistulation can yield a favorable prognosis in dromedary camels. Lesion type, tissue viability, and injury duration are the key factors influencing management choices and overall prognosis for camelid full-thickness abdominal wall injuries.

## 1 Introduction

Abdominal wall injuries in dromedary camels (*Camelus dromedarius*) present a critical clinical challenge in veterinary surgery, frequently resulting from sharp traumatic insults (e.g., wire fencing, corral boundaries), vehicle collisions, or violent bull camel fighting during the breeding season (1–3). In severe cases, these blunt or penetrating forces result in full-thickness disruption of the abdominal tunic and musculature. This degradation is often exacerbated by acute evisceration or the development of chronic, non-healing fistulous tracts. Unlike superficial wounds, full-thickness defects violate the peritoneal barrier, exposing vital organs to intense environmental contamination and placing the patient at immediate risk of septic peritonitis, hypovolemic shock, and death (4–6).

Despite the high socioeconomic and cultural value of dromedaries in rural and intensive production systems across the Middle East, North Africa, and Asia, specialized literature addressing advanced camelid abdominal surgery remains remarkably scarce (4, 7). Historically, large-scale eviscerations in field settings have carried a terminal prognosis, frequently leading to immediate euthanasia. While diverse clinical experiences, retrospective case series, and individual case reports have documented the management of abdominal emergencies and body wall defects in equine and bovine medicine (8–11), equivalent clinical data, specific prognostic indicators, and definitive surgical workflows remain severely scarce for dromedary camels.

From an anatomical perspective, the dromedary camel possesses a highly developed, heavy fibroelastic sheet known as the tunica flava abdominis (12–14). This structure acts as a vital biological tension band that supports an immense visceral volume and complex multi-compartment forestomach kinetics. Furthermore, camels exhibit unique evolutionary adaptations, including specialized fluid-retention capabilities, distinct forestomach fermentation pathways, and cellular tolerance to intense dehydration (15). These traits confer on them distinct hemodynamic resilience to severe trauma compared with other large domestic animals.

Successful surgical management relies on identifying the involved viscera, assessing tissue viability, and identifying secondary complications (13, 16, 17). Because physical palpation is limited by pain and abdominal splinting, diagnostic ultrasonography is an essential adjunct. It defines the margins of the body wall defect, identifies the herniated organs, and detects intra-abdominal fluid, adhesions, or ischemic changes (2, 18).

In other domestic species presenting with full-thickness abdominal wall injuries, successful management has typically relied on intensive surgical debridement coupled with primary layered anatomical closure, tension-relieving suture configurations, or prosthetic mesh reconstruction; however, corresponding clinical outcomes and specific procedural frameworks remain undocumented in dromedary camels. This knowledge gap often leaves field practitioners without evidence-based protocols, resulting in inconsistent surgical interventions or premature euthanasia. To address this gap, this study evaluates the clinical patterns, visceral involvement, surgical treatments protocol, and survival outcomes of 14 dromedary camels presenting with full-thickness abdominal wall injuries complicated by acute evisceration or chronic fistulation.

## 2 Materials and methods

### 2.1 Animals and study design

A retrospective, descriptive study was performed on dromedary camels admitted for abdominal wall trauma at the Department of Surgery, Camel Hospital, Salam Veterinary Group (Qassim, Saudi Arabia) between September 2023 and December 2025. During this 27-month window, 147 camels presented with various forms of abdominal trauma. To isolate high-acuity cases, a strict inclusion criterion was applied: animals had to present with a confirmed full-thickness abdominal wall injury complicated by either acute evisceration or chronic fistulation. Camels exhibiting superficial wounds, isolated cutaneous lacerations, or subcutaneous hernias without complete fascial penetration were excluded from the study population. Applying these parameters yielded a final study cohort of 14 camels (*N* = 14). The study protocol, clinical workflows, and data collection models were reviewed and formally approved by the Institutional Committee of Animal Welfare and Ethics at the Salam Veterinary Group Camel Hospital (Approval No. SVG.26.06.358), ensuring strict adherence to international standards for animal care and clinical research.

### 2.2 Clinical examinations

Upon admission to the hospital, all camels underwent immediate clinical triage and systematic physical examination. Baseline systemic metrics including heart rate, respiratory rate, rectal temperature, mucous membrane color, and capillary refill time (CRT) were recorded to assess hemodynamic stability. Patient demographics, including age, sex, breed, body weight (kg), primary etiology, and the precise duration of the lesion before admission, were systematically recorded. A localized assessment of the abdominal trauma was then performed to define the topographic location (ventral/umbilical midline or ventrolateral), defect dimensions (length and width of the fascial margins), injury classification (evisceration vs. fistulation), and visceral involvement (intestines, rumen, abomasum, or omentum).

### 2.3 Case classification

Cases were categorized into two main mutually exclusive presentation groups based on a strict hierarchical clinical rule. Evisceration was defined as the acute mechanical displacement of structurally intact abdominal viscera (omentum, rumen, abomasum, or intestines) through a full-thickness abdominal wall defect, without luminal breach. Fistulation was defined by the presence of a definitive patent communication between a gastrointestinal lumen and the external skin surface. If a case presented with overlapping pathologies where a visceral segment was both exteriorized through a wall defect and sustained a concurrent luminal rupture/fistula it was systematically classified under the fistulation cohort. This assignment was dictated by the fact that an active gastrointestinal breach introduces distinct microbiological risks and surgical demands (e.g., localized enterectomy or partial visceral wall resection) that fundamentally differentiate it from isolated mechanical evisceration.

Special clinical attention was directed toward characterizing the health of the exposed tissues, evaluating the degree of gross environmental contamination, and documenting secondary complications such as localized necrosis or tissue gangrene. For clinical categorization, localized necrosis was defined as the presence of well-demarcated, avascular, and non-viable fascial or muscular edges that remained structurally firm and odorless. In contrast, tissue gangrene was strictly defined as advanced tissue death complicated by secondary bacterial putrefaction, characterized macroscopically by a soft, liquefying, or shrunken texture, a distinct foul odor, purulent or serosanguinous exudation, and dark green-to-black tissue sloughing. Secondary blowfly larvae infestation (myiasis) was also recorded. When appropriate and safe, a gentle digital exploration of the wound tract was performed using sterile gloves to confirm the depth of fascial disruption and evaluate the integrity of the surrounding musculature.

### 2.4 Ultrasonographic examinations

Ultrasonographic (US) evaluation was performed immediately following initial clinical stabilization in all evisceration and fistulation cases to map subsurface pathology. Sound waves were emitted using a portable diagnostic ultrasound system equipped with a 3.5–7.5 MHz linear array transducer (X5V, SonoScape, Hamburg, Germany). Camels were examined either in a standing position or in lateral recumbency on a padded surgical table, depending on their hemodynamic stability and safety profile. To ensure diagnostic consistency and reduce inter-operator variability, a single experienced veterinary imaging specialist (E. E.) performed all examinations. Before scanning, the target site and surrounding margins were clipped, cleansed, and coupled with an acoustic transmission gel. The ultrasound assessment focused on quantifying the precise borders of the deep abdominal wall defect, identifying the specific herniated visceral segments and evaluating their wall thickness and peristaltic activity, mapping complex topographic relationships between the displaced organs and the fascial margins, and detecting secondary complications.

### 2.5 Surgical treatment

#### 2.5.1 Preoperative preparation

Before initiating general anesthesia, a complete hematological and biochemical profile was analyzed for each patient using automated analyzers to rule out advanced anemia, profound electrolyte imbalances, or systemic organ failure. Preoperative stabilization included aggressive intravenous (IV) fluid resuscitation via a 14-gauge jugular catheter with balanced polyionic crystalloids (lactated Ringer’s solution) administered at a rate of 10–15 mL/kg/h to correct dehydration and hypovolemic shock. Exposed visceral contents were immediately protected from further desiccation and contamination using sterile laparotomy sponges soaked in warm, sterile 0.9% normal saline solution and secured with a temporary external abdominal wrap. A standardized, broad-spectrum preoperative antimicrobial and anti-inflammatory regimen was administered intramuscularly and intravenously: cefquinome (1.5 mg/kg IM; Cobactan 2.5%, MSD Animal Health) and meloxicam (0.5 mg/kg IV; Metacam, Boehringer Ingelheim).

#### 2.5.2 Anesthetic protocol and monitoring

Food was withheld for 24 h and water for 12 h prior to surgery, except in hyperacute, life-threatening evisceration emergencies where immediate surgery superseded fasting. General anesthesia was induced using a total intravenous anesthesia (TIVA) regimen. Camels were premedicated with xylazine HCl (0.2 mg/kg IV; Chanazine 10%, Chanelle). Exactly 10 min following premedication, when profound sedation and head drop were achieved, anesthesia was induced via the rapid bolus injection of ketamine HCl (1.5 mg/kg IV; Cetamin, Agro-Veterinaria). Following recumbency, the trachea was immediately intubated blindly using a cuffed endotracheal tube (size 18–22 mm internal diameter) with the aid of a mouth gag to maintain airway patency. Anesthesia was maintained via intermittent IV boluses of a drip combination (500 mg xylazine and 1,000 mg ketamine added to 1 L of normal saline) titrated to effect (typically 1–2 mL/kg/h). If patients exhibited transient signs of light anesthesia (e.g., nystagmus or tachypnea) during deep dissection, supplemental intravenous boluses of ketamine HCl (0.5 mg/kg IV) were administered. Prior to the first surgical incision, regional preemptive analgesia was achieved by local tissue infiltration around the margins of the body wall defect using 20–40 mL of 2% Lidocaine HCl (Lidocaine 2%, Norbrook) via an inverted-L pattern. Continuous clinical monitoring was performed every 5 min by an anesthetist, tracking the depth of anesthesia via corneal and palpebral reflexes, globe position, respiratory rate and pattern, heart rate (maintained between 35 and 55 beats/min via base-apex ECG), pulse oximetry (SpO₂), and indirect arterial blood pressure measured via an oscillometric cuff placed around the base of the tail.

#### 2.5.3 Surgical technique and reconstruction

Following induction, the animals were secured in lateral recumbency on a heavily padded surgical table to prevent radial nerve paralysis and myopathy. The entire ventral and ventrolateral abdominal field were wide-clipped, shaved, and aseptically prepared using a 10% povidone-iodine scrub (minimum 5-min contact time) followed by a 70% isopropyl alcohol rinse.

For acute evisceration cases, the traumatic defect margins were carefully inspected. Exposed viscera were evaluated for viability based on normal serosal color (pink to dark red), active mesenteric arterial pulsation, and spontaneous or stimulated peristalsis. Exposed loops underwent thorough mechanical debridement to remove macroscopic soil, sand, or feces, followed by copious lavage (6–10 L) with warm, sterile 0.9% saline. Devitalized, ragged fascial borders, necrotic subcutaneous fragments, and compromised skin margins were aggressively excised using sharp dissection back to healthy, actively bleeding tissue zones. Viable viscera were carefully reduced back into the peritoneal cavity.

For chronic fistulation cases, a circumferential fusiform skin incision was made 2 cm away from the fistulous opening. An en bloc radical excision of the entire fistulous tract was performed using sharp dissection down to the level of the abdominal tunic. Dense fibrous adhesions binding the forestomach (C1/C2) or intestinal segments to the internal abdominal wall were meticulously dissected using metzenbaum scissors and digital blunt dissection. Necrotic, heavily scarred, or fistulated visceral segments underwent sharp surgical resection followed by primary closure or hand-sewn end-to-end anastomosis. The visceral repair line was routinely wrapped and reinforced with a pedicle graft of autologous omentum (omentalization) secured with size 2–0 polyglycolic acid in an interrupted pattern to secure the biological seal.

Prior to body wall closure, the entire peritoneal cavity was thoroughly lavaged with 5 liters of warm sterile normal saline solution supplemented with gentamicin sulfate (4.4 mg/kg). Surgical reconstruction of the deep abdominal wall defect was executed using a standardized two-layer anatomical closure technique. Layer 1 (Deep peritoneal seal): First, the isolated peritoneal margins were opposed independently using a continuous suture pattern with Size 1 polyglycolic acid. This step was performed to re-establish a secure peritoneal seal and minimize the risk of postoperative visceral-to-abdominal wall adhesions. Layer 2 (Load-bearing fascial reconstruction): Following peritoneal isolation, a separate, heavy structural layer was placed superficially to reconstruct the primary load-bearing musculofascial tunic. This was achieved by placing a series of interrupted horizontal mattress sutures exclusively through the thick margins of the tunica flava abdominis and external rectus sheath using size 1 polyglycolic acid to provide maximum tension relief against high intra-abdominal forces characteristic of dromedary camels. The overlying subcutaneous tissue layer was closed in a continuous anatomical pattern using size 1 polyglycolic acid to obliterate subcutaneous dead space and reduce seroma formation. Finally, the skin margins were routinely apposed using size 1 polyglycolic acid suture material placed in a continuous Ford interlocking pattern to optimize tension distribution and expedite closure time (13, 19) (Figures 1–5).

**Figure 1:**
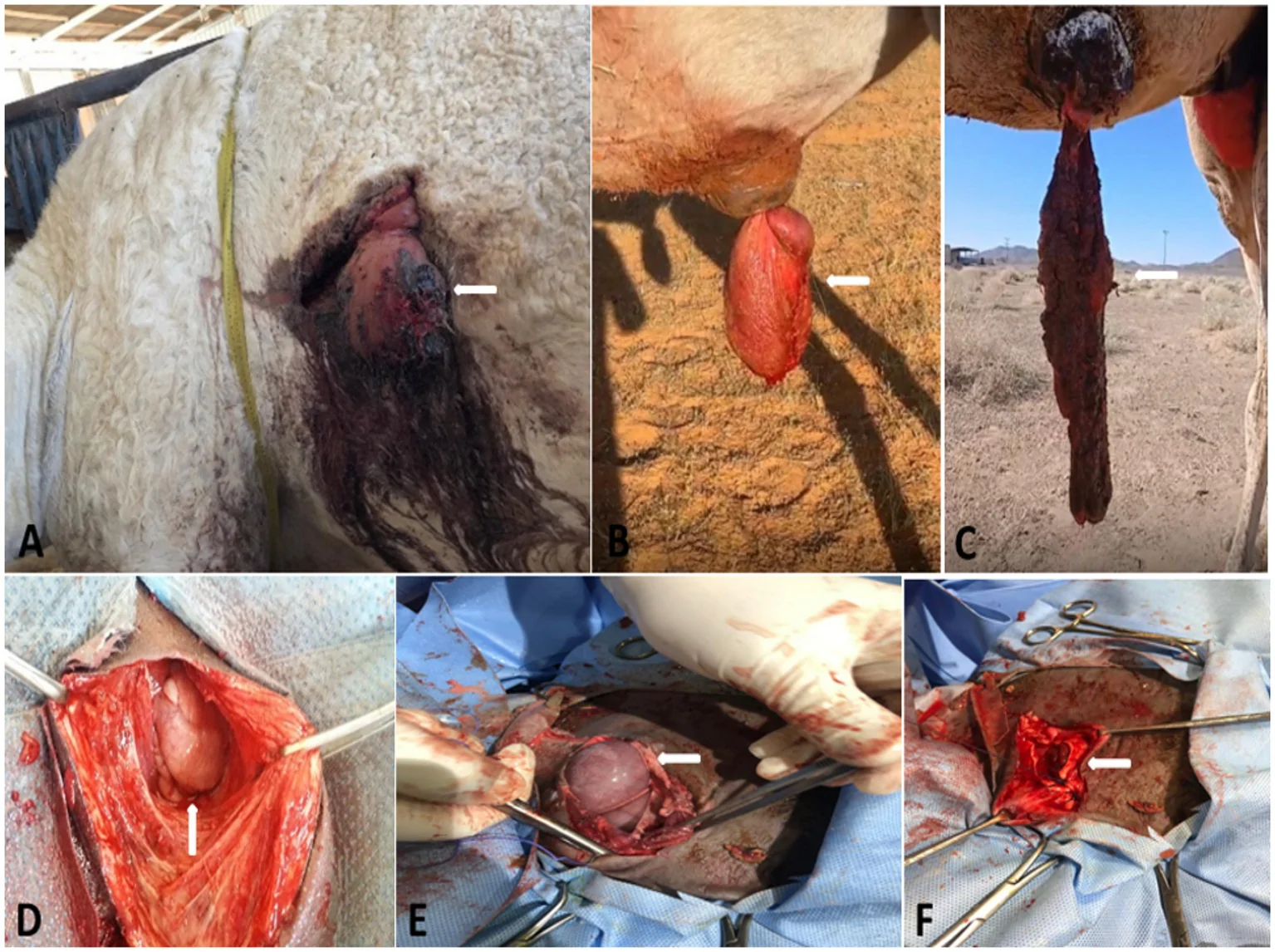
Full-thickness abdominal wall defects with organ evisceration in dromedary camels. Clinical presentations: **(A)** chronic ruminal evisceration (arrow) through a left lateral abdominal wall defect in a 4-year-old camel. **(B)** Ruminal evisceration (arrow) through a ventral/umbilical abdominal wall defect in a 7-year-old camel. **(C)** Chronic abomasal evisceration (arrow) through a ventral/umbilical abdominal wall defect, complicated by severe local infection and tissue necrosis. **(D)** Omental evisceration (arrow) through a ventrolateral defect in a 5-year-old female camel. Intraoperative stages: **(E)** surgical reduction of the eviscerated rumen (arrow) following radical debridement and copious abdominal lavage. **(F)** Double-layer anatomical reconstruction of the deep abdominal wall defect (arrow).

**Figure 2:**
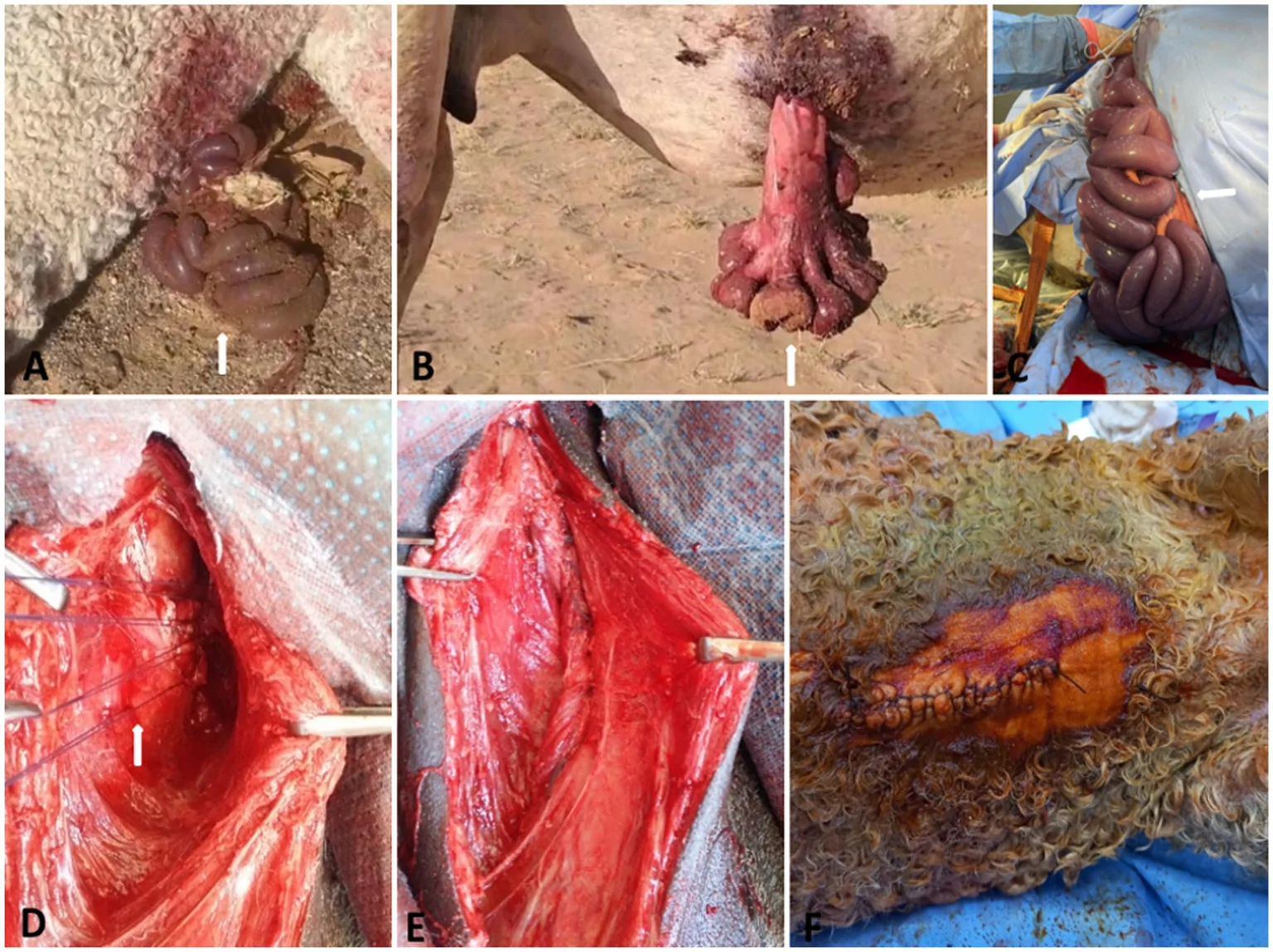
Full-thickness abdominal wall defects with intestinal evisceration and sequential surgical management. Clinical presentations: **(A)** congenital intestinal evisceration (gastroschisis, arrow) through an umbilical defect in a newborn camel calf, complicated by severe local contamination and intestinal gangrene. **(B)** Acute intestinal evisceration (arrow) through a ventrolateral abdominal wall defect in a 6-year-old female camel, presenting 2 days post-injury with marked environmental contamination. Sequential surgical management: **(C)** intraoperative assessment and gentle antiseptic cleansing of the exposed, viable intestinal loops (arrow). **(D)** Reduction of viable intestinal segments into the abdominal cavity following thorough decontamination and peritoneal closure (arrow). **(E)** Mechanical closure of the deep abdominal wall defect following complete reduction of the herniated viscera. **(F)** Final cutaneous apposition using a continuous Ford interlocking skin suture pattern.

**Figure 3:**
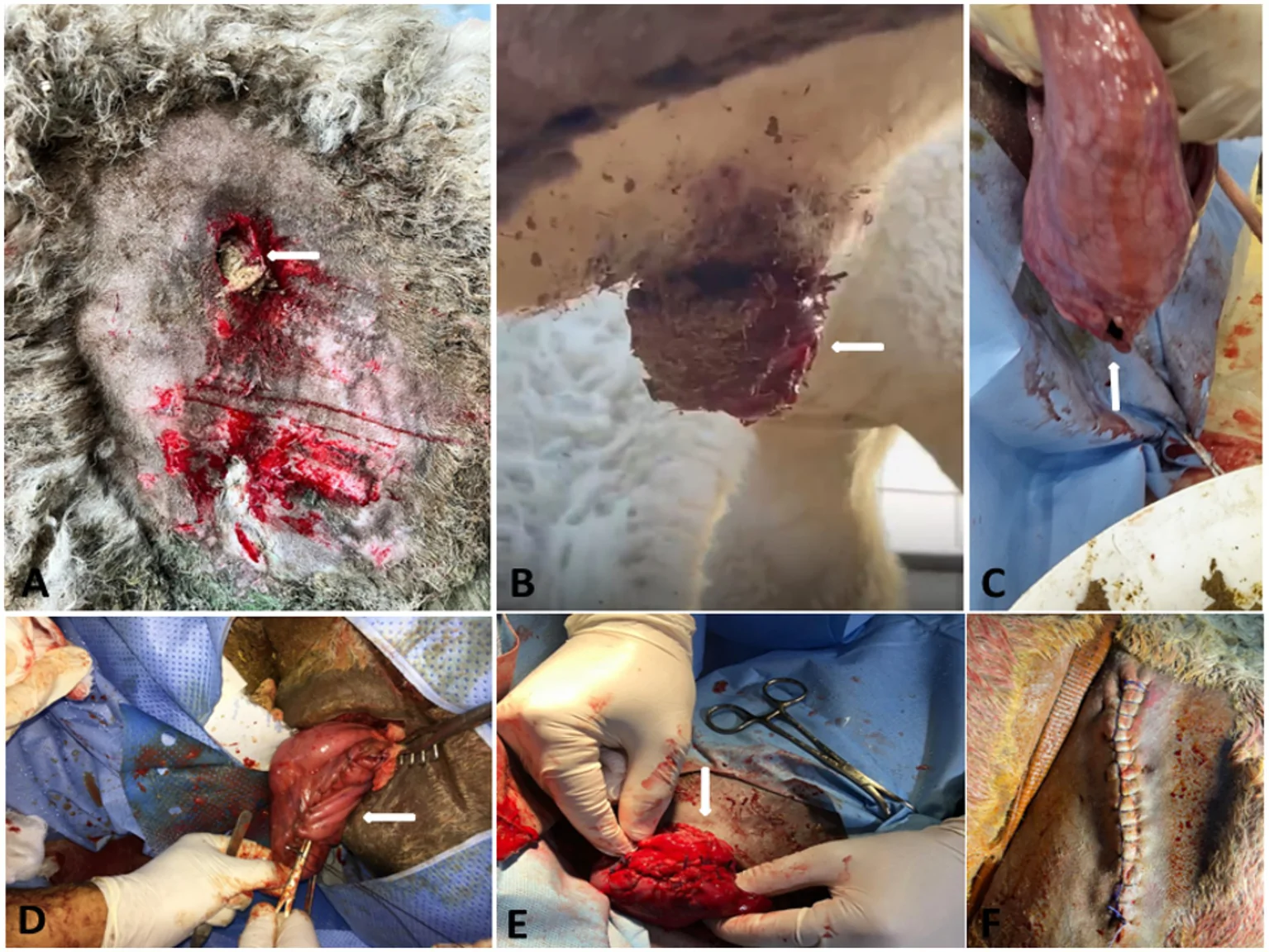
Full-thickness abdominal wall defects complicated by fistulation and surgical repair. Clinical presentations: **(A)** chronic ventrolateral rumino-cutaneous fistula (arrow) characterized by extensive local infection and peripheral tissue necrosis. **(B)** Ventral/umbilical chronic abomasal fistula (arrow) secondary to a neglected umbilical hernia, accompanied by localized tissue degradation. **(C)** Ventrolateral intestinal fistula (arrow) resulting from penetrating trauma from fence wire, presenting 1-day post-injury with minimal regional tissue involvement. Intraoperative stages: **(D)** primary closure of the isolated ruminal fistula (arrow) following surgical debridement and decontamination. **(E)** Direct repair of the intestinal fistula (arrow) after meticulous debridement of the affected loop margins. **(F)** Reconstruction of the abdominal wall defect following anatomical reduction of the repaired fistulated organs.

**Figure 4:**
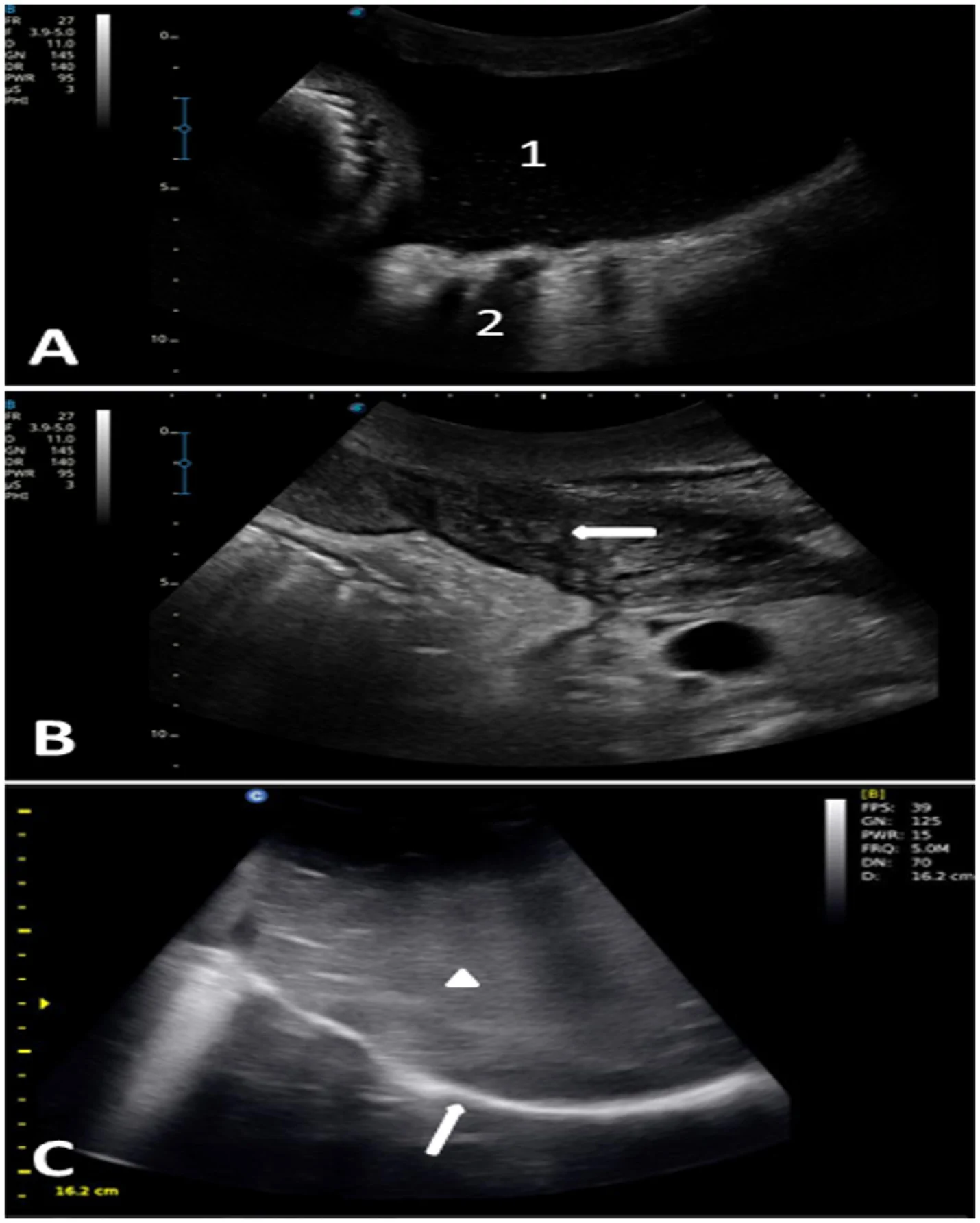
Abdominal ultrasonography of full thickness abdominal wall defect in dromedary camels. **(A)** Ruminal evisceration from abdominal wall defect revealed identification of the rumen cavity (1) and its sacs (2). **(B)** Intestinal evisceration from the abdominal wall defect presented with its longitudinal loop containing ingesta (arrow). **(C)** Abomasal evisceration from abdominal wall defect revealed the thickened hyperechoic abomasal wall (arrow) and heterogeneous contents (arrow head).

**Figure 5:**
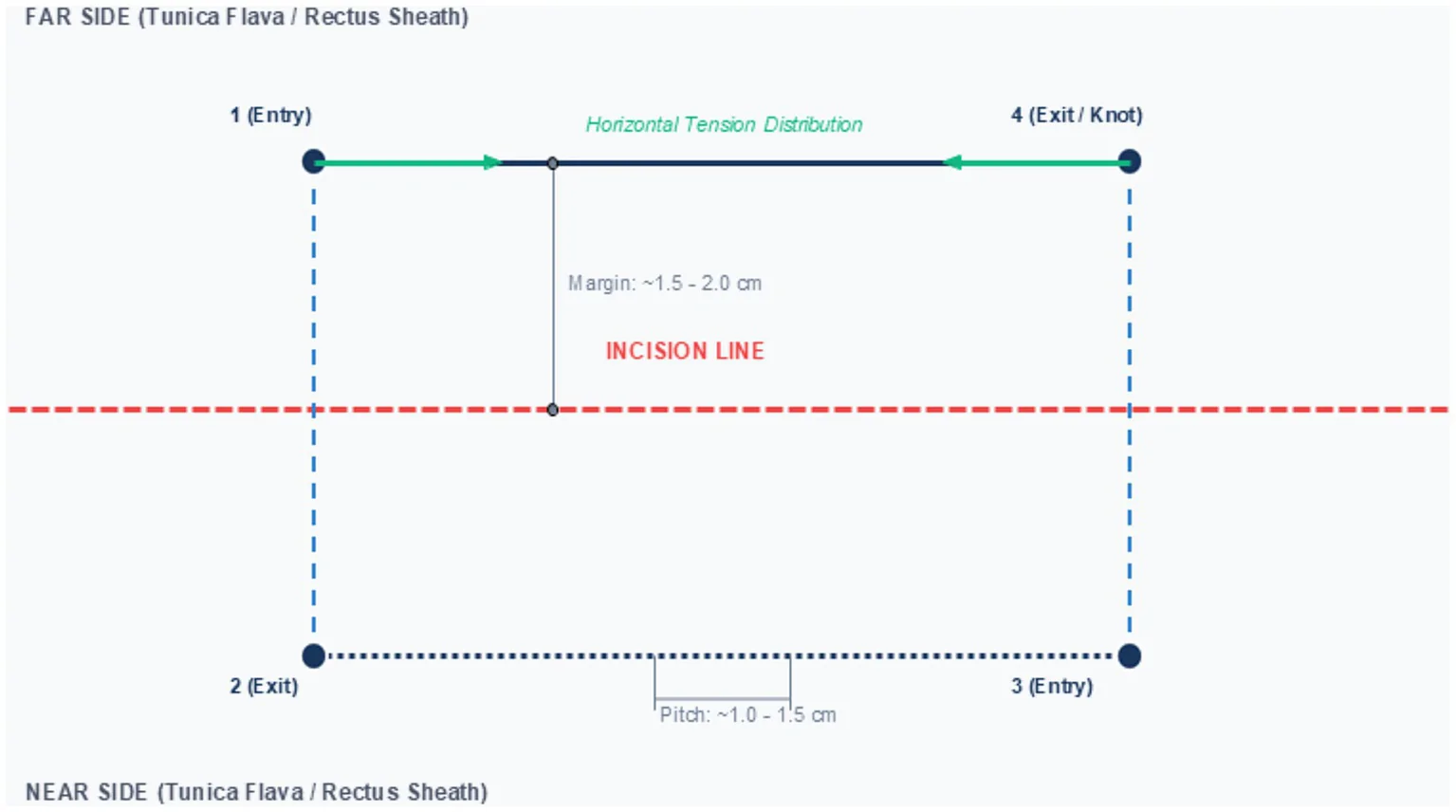
Schematic illustration demonstrating the mechanical placement of the interrupted horizontal mattress suture pattern used for reconstruction of the deep abdominal wall. Suture bites penetrate both the tunica flava abdominis and external rectus sheath as a single unit, with a wide margin (1.5–2.0 cm) from the fascial edge and a parallel loop width of 1.0–1.2 cm, using size 1 polyglycolic acid.

#### 2.5.4 Postoperative management and follow-up

Following the discontinuation of anesthesia, camels were maintained in sternal recumbency in a quiet, deeply bedded recovery box until full locomotor control was regained. Postoperatively, all camels were housed in clean, dry, well-ventilated individual pens to minimize environmental dust contamination. Postoperative management consisted of intensive daily monitoring for systemic attitude, rectally measured core temperature, heart rate, respiratory rate, appetite, and fecal output (consistency and volume). The surgical incision line was inspected twice daily for the first 14 days for signs of regional edema, excessive exudation, localized pain, or premature suture line dehiscence. The preoperative systemic broad-spectrum antimicrobial cefquinome (1.5 mg/kg IM; Cobactan 2.5%, MSD Animal Health) and anti-inflammatory meloxicam (0.5 mg/kg IV; Metacam, Boehringer Ingelheim) therapies were maintained for five consecutive postoperative days to manage postoperative pain and endotoxemia. Topical wound care consisted of daily cleansing of the surrounding skin with a 0.05% chlorhexidine gluconate solution, followed by the application of an overlying oxytetracycline hydrochloride spray barrier paired with a cypermethrin-based insect repellent to prevent myiasis. No systemic abdominal bandage was applied to the abdominal wall due to the anatomical location and patient intolerance. Skin sutures were removed 14 days post-surgery, contingent upon satisfactory cutaneous scar consolidation and the absence of localized fluid accumulation. Following hospital discharge, long-term follow-up was strictly maintained via direct telephone and video-call communication with the owners for a minimum of 4 weeks to monitor complete structural healing, return to production, and rule out late-onset incisional hernia formation.

### 2.6 Statistical analysis

Data were analyzed using descriptive statistics using SPSS software (version 25.0; IBM Corp.) to summarize the clinical features, parameters, and surgical outcomes. Continuous variables, including age and body weight, were evaluated for normality and are expressed as mean ± standard deviation (SD) alongside median and interquartile range (IQR). Categorical variables and percentages are presented as frequencies and proportions relative to their specific clinical presentation group (acute evisceration vs. chronic fistulation). Due to the highly uneven distribution and small sample size of the chronic fistulation cohort (*n* = 3), comparative inferential statistics (e.g., Chi-square, Fisher’s exact test) were not performed, as low statistical power would risk misleading type II errors. Proportions are descriptive and intended to highlight clinical trends between the distinct pathophysiological entities.

## 3 Results

### 3.1 Study population and demographics

Out of 147 dromedary camels admitted with abdominal wall trauma, 14 animals (9.5%) presented with full-thickness injuries complicated by evisceration or persistent fistulation, fulfilling the study’s inclusion criteria. The study cohort presented a mean age of 3.81 ± 3.16 years, with a median age of 4.50 years (IQR: 0.08–6.75 years). The overall mean body weight of the camels was 389.9 ± 175.0 kg, with a median of 450.0 kg (IQR: 327.5–506.3 kg). When stratified by clinical presentation, acute evisceration patients exhibited a mean age of 3.02 ± 3.12 years and a mean body weight of 365.1 ± 189.4 kg (median: 420.0 kg). Conversely, chronic fistulation patients consisted entirely of mature adults, presenting a higher mean age of 6.70 ± 1.15 years and a mean body weight of 480.0 ± 52.9 kg (median: 480.0 kg; Table 1). Female camels predominated the sample population, accounting for 12 out of 14 cases (85.7%), while males comprised only two cases (14.3%). Regarding breed distribution, the Wadeh breed was most frequently affected, representing 11 cases (78.6%), followed by the Sofor breed with three cases (21.4%) (Table 1).

**Table 1:** Comparative features of acute evisceration vs. chronic abdominal wall fistulation in dromedary camels (*n* = 14).

Parameters	Acute evisceration (*n* = 11)	Chronic fistulation (*n* = 3)	Total cohort (*n* = 14)
Frequency (*n* = 14)	(*n* = 11, 78.6%)	(*n* = 3, 21.4%)	(*n* = 14, 100%)
Age (years)	Mean ± SD: 3.02 ± 3.12 Median (IQR): 3.00 (0.08–5.50)	Mean ± SD: 6.70 ± 1.15 Median (IQR): 6.50 (5.85–7.35)	Mean ± SD: 3.81 ± 3.16 Median (IQR): 4.50 (0.08–6.75)
Body weight (Kg)	Mean ± SD: 365.1 ± 189.4 Median (IQR): 420.0 (240.0–495.0)	Mean ± SD: 480.0 ± 52.9 Median (IQR): 480.0 kg (445.0–515.0)	Mean ± SD: 389.9 ± 175.0 Median (IQR): 450.0 (327.5–506.3)
Sex	Female: (*n* = 9, 81.8%) Male: (*n* = 2, 18.2%)	Female: (*n* = 3, 100.0%) Male: (*n* = 0, 0.0%)	Female: (*n* = 12, 85.7%) Male: (*n* = 2, 14.3%)
Breed	Wadeh: (*n* = 9, 81.8%) Sofor: (*n* = 2, 18.2%)	Wadeh: (*n* = 2, 66.7%) Sofor: (*n* = 1, 33.3%)	Wadeh: (*n* = 11, 78.6%) Sofor: (*n* = 3, 21.4%)
Presentation time	Early presentation (≤ 2 days, *n* = 10, 90.9%) Delayed (1–2 weeks, *n* = 1, 9.1%)	Delayed (1–2 weeks, *n* = 1, 33.3%) Chronic (1–3 months, *n* = 2, 66.7%)	Early presentation (≤ 2 days, *n* = 10, 71.4%) Delayed (1–2 weeks, *n* = 2, 14.3%) Chronic (1–3 months, *n* = 2, 14.3%)
Primary etiology	Congenital (Gastroschisis, *n* = 2, 18.2%) Traumatic (fences, etc., *n* = 6, 54.6%) Hernia rupture (*n* = 3, 27.3%)	Traumatic (fences, etc., *n* = 2, 66.7%) Hernia rupture (*n* = 1, 33.3%)	Congenital (Gastroschisis, *n* = 2, 14.3%) Traumatic (fences, etc., *n* = 8, 57.1%) Hernia rupture (*n* = 4, 28.6%)
Topographic classification	Ventral midline (*n* = 5, 45.5%) Ventrolateral wall (*n* = 6, 54.5%)	Ventral midline (*n* = 2, 66.7%) Ventrolateral wall (*n* = 1, 33.3%)	Ventral midline (*n* = 7, 50.0%) Ventrolateral wall (*n* = 7, 50.0%)
Visceral involvement	Intestines (*n* = 6, 54.5%) Rumen (*n* = 3, 27.3%) Abomasum (*n* = 1, 9.1%) Omentum (*n* = 1, 9.1%)	Intestines (*n* = 1, 33.3%) Rumen (*n* = 1, 33.3%) Abomasum (*n* = 1, 33.3%)	Intestines (*n* = 7, 50.0%) Rumen (*n* = 4. 28.6%) Abomasum (*n* = 2, 14.3%) Omentum (*n* = 1, 7.1%)
Pathological features	Raw visceral displacement, severe acute tissue tearing, high environmental debris/fecal contamination, acute edema, or ischemic necrosis risk.	Fibrous encapsulation, well-established draining tracts, dense internal visceral adhesions to body wall tunics, and localized focal necrosis.	
Local complications	Gross contamination (*n* = 11, 100%)	Localized infection & necrosis (*n* = 2, 66.7%) Myiasis, necrosis & gangrene (*n* = 1, 33.3%)	Gross contamination (*n* = 11, 78.6%) Localized infection & necrosis (*n* = 2, 14.3%) Myiasis, necrosis & gangrene (*n* = 1, 7.1%)
Ultrasonographic findings	Complete muscular tunic disruption; free peritoneal fluid; visible peristalsis or echo-profile of acute herniated loops (intestines/rumen/abomasum).	Hyperechoic, heavily thickened fibrous margins mapping complex subsurface fistulous paths; dense organ adhesions linked to the fascial borders.	
Surgical treatment	Hyperacute triage, intensive polyionic fluid resuscitation, immediate raw visceral coverage, active lavage, mass double-layer tension closure.	En-bloc surgical fistulectomy, meticulous dissection/adhesiolysis of visceral organs, sharp debridement to healthy tissue margins, layered reconstruction.	
Treatment outcomes	Died pre-treatment (*n* = 1, 9.1%) Recovered & Discharged (*n* = 7, 63.6%) Died post-treatment (sepsis) (*n* = 3, 27.3%)	Recovered & Discharged (*n* = 2, 66.7%) Died post-treatment (sepsis) (*n* = 1, 33.3%)	Died pre-treatment (*n* = 1, 7.1%) Recovered & Discharged (*n* = 9, 64.3%) Died post-treatment (*n* = 4, 28.6%)
Postoperative complications	Localized incisional edema (*n* = 6, 54.5%) Transient anorexia/ depression (*n* = 5, 45.5%) Superficial wound exudation (*n* = 2, 18.2%) Transient fecal output alterations (*n* = 4, 36.4%) Superficial skin dehiscence (*n* = 1, 9.1%) Severe postoperative sepsis (*n* = 3, 27.3%)	Localized incisional edema (*n* = 1, 33.3%) Transient anorexia/ depression (*n* = 1, 33.3%) Superficial wound exudation (*n* = 2, 66.7%) Transient fecal output alterations (*n* = 0) Superficial skin dehiscence (*n* = 1, 33.3%) Severe postoperative sepsis (*n* = 1, 33.3%)	Localized incisional edema (*n* = 7, 50.0%) Transient anorexia/ depression (*n* = 6, 42.9%) Superficial wound exudation (*n* = 4, 28.6%) Transient fecal output alterations (*n* = 4, 28.6%) Superficial skin dehiscence (*n* = 2, 14.3%) Severe postoperative sepsis (*n* = 4, 28.6%)

### 3.2 Injury classification and topographic features

Anatomical distribution of the full-thickness body wall defects was equally split between the ventral midline zone (*n* = 7, 50.0%) and the ventrolateral abdominal wall (*n* = 7, 50.0%). Acute evisceration was identified as the primary presentation type in 11 camels (78.6%), while chronic, persistent fistulation was documented in three cases (21.4%). Clinical appearance of each ventral (Figures 1A,D,E,F, 2C–E, 3A,C–F) and ventrolateral abdominal wall (Figures 1B,C, 2A,B,F, 3B) lesion was showed in Figures 1–3. Acute evisceration lesions were presented in Figures 1, 2, while chronic fistulation lesions were presented in Figure 3.

The primary etiology varied across three main categories: direct external trauma (sharp objects, including farm fences and corral boundaries) were the most common cause (*n* = 8, 57.1%), secondary hernia rupture (incisional or ventral hernia progression) accounted for four cases (28.6%), and congenital abdominal wall defects (complete gastroschisis) was observed in two neonatal calves (14.3%).

### 3.3 Chronicity, pathological changes, and visceral involvement

The timing between injury occurrence and hospital presentation differed significantly. Most animals presented within an early presentation window (1–2 days post-injury; *n* = 10, 71.4%). Delayed presentations (1–2 weeks post-injury) were observed in two cases (14.3%), and chronic presentations (1–3 months post-injury) accounted for the remaining two camels (14.3%).

Exposed abdominal contents varied based on the defect’s location. The intestines were the most frequently involved viscera (*n* = 7, 50.0%), followed by the rumen (*n* = 4, 28.6%), abomasum (*n* = 2, 14.3%), and the omentum alone (*n* = 1, 7.1%).

Pre-operative local evaluations revealed advanced local pathology across all cases: gross contamination severe dirt, debris, and fecal contamination were noted in 11 cases (78.6%). Localized infection and tissue necrosis (deep fascial infection and tissue devitalization were isolated in two cases (14.3%). Advanced tissue necrosis, gangrene, and secondary screwworm fly larvae infestation (myiasis) was present in one case (7.1%).

### 3.4 Ultrasonographic findings

In cases of full thickness abdominal wall injuries presenting with evisceration, ultrasonographic examination enabled identification and confirmation of the affected abdominal viscera. It also allowed assessment of their relationship with the abdominal wall defect. Ruminal evisceration from abdominal wall defect was easily identified by US features of the rumen cavity and its sacs (Figure 4A). Intestinal evisceration from the abdominal wall defect presented with its echogenic longitudinal loop containing hypoechoic ingesta (Figure 4B). In addition, the abomasal evisceration from abdominal wall defect revealed the thickened hyperechoic abomasal wall and heterogeneous contents (Figure 4C).

### 3.5 Surgical treatment outcomes

A cross-comparison between presentation timing and final clinical outcomes revealed distinct survival profiles.

#### 3.5.1 Acute evisceration cohort (*n* = 11)

Of the 11 dromedary camels presenting with acute abdominal wall evisceration, one patient (9.1%), (1/11) succumbed to severe systemic shock and gross contamination prior to surgical intervention. The remaining 10 camels underwent emergency surgical intervention involving mass double-layer tension closure. Following surgery, (63.6%) (7/11) of the total cohort survived and were successfully discharged from the hospital. Conversely, (27.3%) (3/11) of the camels developed rapid hemodynamic collapse and hyperacute systemic sepsis within 24 to 48 h post-injury, which resulted in their death.

#### 3.5.2 Chronic fistulation cohort (*n* = 3)

All 3 camels presenting with chronic abdominal wall fistulation underwent extensive en-bloc surgical fistulectomy, meticulous adhesiolysis, and layered abdominal wall reconstruction. Postoperative recovery and successful hospital discharge were achieved in (66.7%) (2/3) of the cases. One camel (33.3%), (1/3) developed uncontrollable severe sepsis on postoperative day 4, secondary to chronic focal necrosis following extensive, prolonged surgical dissection and adhesiolysis of dense fibrous tracts attaching the visceral organs to the abdominal wall tunics.

### 3.6 Postoperative complications

Postoperative monitoring revealed varying degrees of localized and systemic complications across the 14 cases, heavily influenced by the pre-existing environmental and fecal contamination of the injuries (Table 1).

Minor and Transient Complications: localized ventral or ventrolateral incisional edema along the suture lines was the most frequent minor complication, observed in 50.0% (7/14) of the total cohort, predominantly affecting the acute evisceration cases (*n* = 6/11). Transient anorexia and a depressed systemic attitude during the first 48–72 h post-surgery occurred in 42.9% (6/14) of the camels. Superficial wound exudation confined to the subcutaneous tissues occurred in 28.6% (4/14) of cases. Mild, short-term alterations in fecal output (soft feces or reduced volume) were noted exclusively in camels that underwent surgical manipulation of intestinal loops (28.6%, 4/14). All minor complications resolved via conservative management, systemic anti-inflammatories, or second-intention cutaneous healing.

Major and Fatal Complications: Severe systemic sepsis was the primary major complication, resulting in postoperative death in 28.6% (4/14) of the treated animals. This catastrophic outcome occurred in 27.3% (3/11) of the acute evisceration cases and 33.3% (1/3) of the chronic fistulation cases, directly correlating with severe pre-operative tissue necrosis, visceral gangrene, or chronic internal adhesions. No mechanical breakdown of the deep abdominal wall closure (incisional dehiscence) or relapse of evisceration was observed in the remaining animals that survived past the initial postoperative week.

## 4 Discussion

Full-thickness abdominal wall injuries associated with acute evisceration or chronic fistulation are uncommon but potentially life-threatening surgical emergencies in dromedary camels (1, 4). These lesions pose considerable diagnostic and therapeutic challenges because of the risk of profound visceral contamination, advanced tissue devitalization, septic complications, and abdominal wall reconstruction failure (3, 7). To the authors’ knowledge, this study represents the first retrospective case series to explicitly bifurcate and analyze the distinct clinical, diagnostic, and surgical behaviors of acute evisceration (*n* = 11) versus chronic abdominal wall fistulation (*n* = 3) in this species.

The overall recovery and discharge rate of 64.3% (*n* = 9/14) achieved across the entire cohort indicates that favorable outcomes are attainable when these complex cases are managed with structured surgical strategies. However, a closer look at the data reveals that success is highly dependent on the presentation archetype. Acute evisceration cases demanded rapid emergency stabilization and immediate containment of contamination, while chronic fistulation cases required technically demanding anatomical reconstruction, extensive debridement of dense fibrosis, and meticulous adhesiolysis (2, 20, 21).

A notable finding in our series was the successful execution of primary abdominal wall reconstruction without the need for prosthetic mesh implantation in 11 out of 13 surgical cases. The favorable survival rate achieved despite a 78.6% overall prevalence of gross environmental contamination is heavily supported by the dromedary’s unique evolutionary and physiological tolerance to massive fluid shifts and prolonged visceral exposure. From an anatomical standpoint, the camel’s abdominal wall features a phenomenally well-developed tunica flava abdominis. This fibroelastic tunic provides substantial tensile strength and excellent suture retention capabilities. This mechanical durability is particularly critical given the massive physical forces involved; the mature animals in this cohort reached body weights up to 570 kg (with chronic fistulation cases mean weight of 480.0 ± 52.9 kg), creating immense baseline intra-abdominal pressure (14, 22, 23). The use of large-gauge (size 1) polyglycolic acid absorbable sutures placed in a heavy, tension-relieving horizontal mattress pattern effectively distributed this substantial massive visceral load. This technique anchored the tunica flava and underlying peritoneum in secure layers, minimizing the risk of suture pull-through or incisional dehiscence. Avoiding synthetic mesh implantation may also reduce the risk of persistent foreign-body reactions, chronic sinus tract formation, and catastrophic implant-associated infections, particularly in contaminated or dirty surgical fields where biofilm-forming bacteria are present (22–24).

While synthetic monofilament materials like nylon are traditionally preferred in contaminated fields to reduce bacterial adherence, polyglycolic acid was selected for cutaneous closure in this cohort primarily due to the logistics of pastoral camel management, where patient recapture for suture removal is frequently unfeasible (22–24). To mitigate the risk of bacterial wicking inherent to multifilament materials in contaminated abdominal injuries, aggressive local tissue debridement, intensive antiseptic irrigation, and postoperative topical protection were strictly enforced.

The favorable deep muscular healing outcomes observed in this cohort suggest unique species-specific physiological adaptations in the dromedary camel. First, the primary structural layer of the camel’s ventral abdomen is dominated by the tunica flava abdominis a highly specialized, thick, elastic fibrous tunic. This dense extracellular matrix provides a remarkably robust mechanical anchor that holds heavy, large-gauge suture strands securely without the focal ischemic necrosis or cheese-wire structural slicing often seen in the friable rectus abdominis sheath of horses. Second, *Camelus dromedarius* exhibits an exceptionally aggressive peritoneal mesothelial response (13, 14). Upon injury or surgical intervention, the camel peritoneum rapidly mobilizes dense fibrin sheets that quickly encapsulate the internal suture line, effectively walling off microscopic bacterial contaminants before a clinical abscess can mature. Coupled with the camel’s resilient innate cellular immune profile, these factors may render the deep abdominal wall more tolerant of multifilament materials than other large animal species (19, 22).

The anatomical distribution of full-thickness defects was equally split between the ventral midline (50.0%) and the ventrolateral wall (50.0%). This distribution differs from classic blunt-force traumatic abdominal hernias in camels, which predominantly involve the flank or ventrolateral tunics without skin breach (12, 13). The high frequency of intestinal (50.0%) and ruminal (28.6%) involvement underscores the high-energy nature of these injuries and explains the acute vulnerability to septic peritonitis. Trauma caused by sharp environmental fixtures (e.g., farm fences, corral boundaries) was the leading driver of these lesions (57.1%), followed by secondary hernia rupture (28.6%). The latter underlines the critical importance of correcting simple hernias early before they undergo catastrophic fascial fatigue or entrapment (9, 11, 25).

Congenital defects presented a completely separate clinical and ethical challenge, isolated exclusively as complete gastroschisis in two neonatal calves (14.3%) within Group A. The inclusion of these neonates structurally skewed the overall demographic data, accounting for the low minimum age (0.08 years) and the highly variable body weight standard deviation (365.1 ± 189.4 kg) observed in the acute evisceration cases. Congenital gastroschisis represents an ultra-emergency where hours dictate the boundary between simple visceral reduction and mortality. These neonates suffer from rapid hypothermia, severe tissue desiccation, and progressive gangrene of exposed loops. Surgeons must prioritize these cases for immediate surgical closure to exploit the sterile nature of birth before environmental exposure triggers irreversible shock (4, 6, 19).

The age distribution of affected animals indicates that these conditions may occur throughout life, from neonatal calves to mature adults (3, 10). The demographic clustering toward female camels (85.7%) and the Wadeh breed (78.6%) does not indicate a true biological or genetic susceptibility to body wall failure. Instead, it reflects the hospital’s local population dynamics and the high economic, reproductive, and cultural value assigned to female Wadeh camels in Saudi Arabia, which drives owners to seek advanced veterinary care (2, 11).

Our surgical outcomes emphasize several cardinal principles. First, aggressive debridement back to active, bleeding fascial margins is mandatory for securing the primary closure lines. Second, multi-liter, warm sterile saline lavages supplemented with gentamicin sulfate (4.4 mg/kg) are essential for mitigating bacterial load. Third, wrapping gastrointestinal repair lines with autologous omentum (Omentalization). While omental reinforcement is typically avoided in equine laparotomies to prevent adhesion-related entrapment, omentalization is widely utilized in ruminant, camelid, and small animal surgery to safeguard compromised gastrointestinal closures (5, 9, 20). The physiological rationale rests on the omentum’s dense vascularity, rich lymphatic plexuses, and high expression of angiogenic factors, which collectively accelerate tissue healing, provide local vascularization, delivers rich immune factors, and form a rapid physical barrier against anastomotic leakage in heavily contaminated fields (13, 19).

The core finding of this study is the profound impact of lesion chronicity on tissue viability and surgical approach. Acute evisceration cases were characterized by immediate structural emergency, raw visceral displacement, and severe contamination, yet they retained highly pliable tissue planes that allowed for relatively straightforward primary closure. Conversely, chronic fistulation cases presented with a consolidated pathological triad of extensive tissue necrosis, chronic infection, and severe localized myiasis (7.1%) (2, 8, 11).

While chronic cases have established tracts prone to persistent local drainage, acute cases are more prone to massive fluid shifts and broad incisional edema due to raw, extensive trauma. Unlike enterocutaneous fistulae involving the small intestine, which are commonly associated with rapid, severe fluid and electrolyte disturbances due to the high daily volume of proximal intestinal secretions and the disruption of downstream fluid reabsorption, fistulae involving the stomach compartments or large bowel tend to present with a more gradual, manageable metabolic timeline (4, 5, 9). Remarkably, camels with chronic fistulation exhibited surprising systemic stability at admission. This clinical tolerance is attributable to the fact that these chronic fistulae predominantly involved the forestomach (C1/C2 compartments), where firm, mature adhesions had securely anchored the viscus directly to the margins of the abdominal defect (2, 11). These dense fibrous adhesions effectively localized the leakage to the fistulous tract, preventing diffuse, catastrophic peritoneal contamination. However, this localized protection came at a high surgical cost, requiring complete en bloc fistulectomy and extensive, high-risk adhesiolysis to free the organs before layer-by-layer closure could be attempted (8, 10, 11).

The stratification of our study population revealed distinct clinical courses, complication profiles, and localized pathologies between dromedary camels presenting with acute evisceration versus those with chronic fistulation. In the acute evisceration cohort (*n* = 11), the presentation was predominantly characterized by hyperacute trauma (54.6%) or congenital gastroschisis (18.2%) affecting younger animals (mean age 3.02–3.12 years). The high incidence of localized incisional edema (54.5%) and transient alterations in fecal output (36.4%) in this group can be attributed to the raw visceral displacement, acute tissue tearing, and high environmental or fecal debris contamination associated with sudden wall disruption (5, 7). The transient fecal output alterations likely stem from immediate neurogenic or inflammatory ileus secondary to manual handling of the raw intestines or rumen during hyperacute triage and mass double-layer tension closure (8, 9). Furthermore, the acute mortality rate (27.3%) post-treatment underscores the systemic shock and risk of rapid, overwhelming ischemic necrosis or sepsis inherent to open, gross environmental exposure of abdominal viscera.

Conversely, the chronic fistulation cohort (*n* = 3) comprised older (mean age 6.70–1.15 year), heavier animals (mean weight 480.0–52.9 kg) with well-established pathologies resulting from delayed care of historical injuries. Surgically, these cases demanded en-bloc fistulectomy, extensive debridement to healthy margins, and meticulous adhesiolysis due to dense internal visceral adhesions to the body wall tunics (10, 11). Despite the extensive visceral manipulation required to clear these adhesions, no transient fecal output alterations were observed (0%). This suggests that chronic viscera may possess a degree of local tolerance or that the localized nature of the inflammation limits the widespread neurogenic ileus seen in acute settings. However, the compromised, ischemic, and fibrotic nature of the chronic tissue beds significantly heightened localized healing challenges, as evidenced by a (66.7%) rate of superficial wound exudation (9, 11). The single post-treatment death in this cohort (33.3%) resulted from severe sepsis, highlighting that despite fibrous encapsulation, subsurface focal necrosis and chronic localized infections remain highly hazardous during extensive abdominal wall reconstruction.

The relatively stable systemic condition observed in the camels presenting with chronic fistulation in this cohort is noteworthy, yet it aligns closely with the broader pathophysiological principles documented across other large domestic herbivores. In veterinary literature, when an abdominal wall defect or visceral perforation fails to cause hyperacute, fatal peritonitis, the mature peritoneal response frequently sequesters the insult. This phenomenon is well-documented in adult cattle and calves (2, 11) suffering from chronic rumino-cutaneous or abomaso-cutaneous fistulae secondary to neglected umbilical hernias or penetrating trauma, where patients routinely maintain a normal appetite, stable vital signs, and standard systemic attitude despite a persistent draining tract. Similarly, in equine medicine (8, 9), while acute intestinal exposure is notoriously catastrophic, cases of chronic enterocutaneous fistulae managed under delayed protocols demonstrate remarkable systemic compensation once mature fibrous adhesions have completely walled off the peritoneal cavity from environmental exposure.

Our findings demonstrate that *Camelus dromedarius* utilizes a highly effective variation of this protective peritoneal isolation mechanism. This further underscores that while acute evisceration requires hyperacute metabolic resuscitation, chronic fistulation in camels much like in cattle and horses presents primarily as a localized mechanical and reconstructive challenge rather than an immediate systemic crisis.

Ultimately, mortality in this series was dictated by delayed referral and advanced systemic collapse rather than the morphological classification of the injury itself (5, 20). The animal that died during triage (7.1%) and the four that succumbed to septic peritonitis postoperatively (28.6%) all shared a common denominator of prolonged exposure, advanced tissue devitalization, or septic shock at admission (6, 17).

While clinical evaluation remains the cornerstone of triage, diagnostic ultrasonography performed by an experienced imaging specialist proved to be an invaluable adjunctive tool. It allowed for the accurate mapping of subsurface fascial defects, determined whether the eviscerated structures were intestinal loops (showing echogenic margins and fluid-filled cores) or forestomach walls, and identified the extent of deep adhesions or fluid pockets in chronic tracts (25–27). Ultrasonography should therefore be considered mandatory for planning the surgical approach in both acute and chronic presentations.

We acknowledge several limitations in this study, including its retrospective field nature, the lack of long-term follow-up beyond 4 weeks, and a small sample size (*n* = 14) that precludes robust inferential statistical analysis of prognostic indicators. Our final outcome profiles are heavily constrained by the marked clinical heterogeneity of the study cohort, including traumatic injuries, hernia rupture, and congenital gastroschisis, as well as the heterogeneity of eviscerated/fistulated viscera. Future prospective, randomized trials with extended geographic tracking are required to definitively determine the long-term success rates of this two-layer anatomical reconstruction, to establish the definitive gold-standard suture material for camel laparotomies under field contamination, and to utilize uniform, single-organ inclusion parameters to isolate true prognostic predictors for abdominal wall failure in *Camelus dromedarius*. Nevertheless, given the scarcity of literature on advanced camel laparotomy and emergency body wall reconstruction, these data provide crucial, practical insights for large-animal surgeons operating under both hospital and field conditions.

## 5 Conclusion

Full-thickness abdominal wall injuries in dromedary camels are highly challenging conditions that must be clearly separated into acute evisceration and chronic abdominal wall fistulation. The overall recovery and discharge rate (64.3%) achieved in the present study indicates that favorable outcomes can be obtained for complex abdominal wall injuries in dromedary camels through early surgical intervention, aggressive debridement, and robust double-layer primary closure anchoring the tunica flava abdominis without the necessity for synthetic mesh implants. However, the drivers of success differ substantially by timeline: acute eviscerations require hyperacute stabilization to combat uncontained visceral shock, whereas chronic fistulations represent a hemodynamically stable, survivor-filtered population where favorable outcomes are contingent upon meticulous surgical adhesiolysis rather than immediate systemic resuscitation. Prognosis is heavily dictated by presentation lag, tissue necrosis, and systemic sepsis, highlighting the need for prompt clinical referral and the use of diagnostic ultrasonography to optimize surgical planning.

## Data Availability

The original contributions presented in the study are included in the article/supplementary material, further inquiries can be directed to the corresponding author.
